# Constitutional mutations of cancer susceptibility genes in the health workers professionally exposed to cytostatic drugs

**DOI:** 10.1186/1897-4287-9-S2-A2

**Published:** 2011-06-01

**Authors:** H Janiszewska, K Szmyd, A Bąk, M Schab, M Pilarska, A Junkiert-Czarnecka, O Haus

**Affiliations:** 1 Department of Clinical Genetics, Collegium Medicum, Nicolaus Copernicus University, Bydgoszcz, Poland; 2 Department of Pediatric Bone Marrow Transplantation, Oncology and Hematology, Medical University, Wroclaw, Poland; 3 Department of Hematology, Oncology and Bone Marrow Transplantation, Medical University, Wroclaw, Poland

## Background

Many studies on the genetic background of common neoplastic diseases indicate that they may be the result of hereditary mutations in cancer susceptibility genes, such as *BRCA1*, *CHEK2*, *CDKN2A*, *NOD2*, *NBS1*. The cancer occurrence in genetically predisposed mutation carriers may be a matter of life expectancy, efficiency of the immunological system, and cumulative action of toxic environmental agents. Most cytostatic drugs used in the treatment of cancers in oncological wards have mutagenic and carcinogenic activity. The exposure to them is difficult to avoid by the medical staff in their daily work during preparation and administration of drugs as well as during the contact with patients (physical examination, nursing care).

## Aim

Investigations of nine Polish founder mutations: *BRCA1*(5382insC, 300T/G, 4153delA), *CHEK2*(1100delC, I157T, IVS2+1G→A), *CDKN2A*(A148T), *NOD2*(3020insC) and *NBS1*(657del5) in healthcare personnel of oncohematological ward, with respect to the age of cancer diagnosis, a cancer family history and a number of work years in contact with cytostatics.

## Tested persons

75 persons (55 nurses, 16 physicans and 4 laboratory workers) exposed to cytostatic drugs in their daily work. In 10 of them during the period of employment in the oncohematological ward a cancer was diagnosed: in 6 women breast cancer (BrCa), in 2 persons melanoma, in one person secondary MDS-related AML, and in one non-Hodgkin lymphoma (NHL). The mean number of work years in contact with cytostatics, till the time of molecular studies for the whole analyzed group, was 13.1 years (range 1-29 years) and for 10 people with cancer diagnosis 15.2 years (range 7-27 years).

## Methods

Mutations in *BRCA1* (5382insC, 300T/G, 4153delA), *CHEK2* (1100delC, I157T, IVS2+1G→A), *CDKN2A* (A148T), *NOD2* (3020insC) and *NBS1* (657del5) were detected in DNA from peripheral blood by RFLP-PCR and ASO-PCR techniques.

## Results

A constitutional mutation was found in 24% persons: *NOD2*(3020insC) in 10.7%, *CDKN2A*(A148T) in 6.7%, *CHEK2*(I157T) in 5.3%, and *CHEK2*(IVS2+1G→A) in 1.3%. The frequency of *NOD2*(3020insC), *CDKN2A*(A148T), *CHEK2*(I157T) and *CHEK2*(IVS2 1→G) was significantly higher than in general Polish population (10.7% vs. 7.3%; 6.7% vs. 3.5%; 5.3% vs. 4.8% and 1.3% vs. 0.48%, respectively). Among 10 persons with cancer in 6 (60%) a mutation was revealed: in 5 out of 6 women with BrCa and in one man out of two persons with melanoma. In the group of women with BrCa a 50%, 16.7% and 16.7% frequency of *NOD2*(3020insC), *CDKN2A*(A148T), and *CHEK2*(I157T) was found, respectively. The *NOD2*(3020insC) variant allele was found to be associated with the increased risk of BrCa (OR=12.6; p=0.01) in this group. The mean age of cancer onset in mutation carriers was 3.5 years older than in non-carriers (43.0 vs. 39.5 years). 72% of mutation carriers originated from families with at least one cancer in a close relative.

## Comments

These are the first study on the impact of occupational exposure to cytostatics of persons with constitutional mutations in cancer susceptibility genes to cancer development. The results show an association between daily exposure to cytostatics and the increased risk of occurrence of cancer in genetically predisposed healthcare personnel of the oncohematology ward. However, we suspect that the hereditary mutation may cause delay of cancer onset in these persons. To confirm our supposition, it is necessary to examine a larger group of healthcare workers of oncology wards.

